# Effects of sedative practices on grief in the spouse of a patient who has died of cancer. An international systematic review

**DOI:** 10.3389/fpsyg.2025.1603194

**Published:** 2026-01-12

**Authors:** Yasmine Chemrouk, Livia Sani, Marthe Ducos, Pascal Gauthier, Marie-Frédérique Bacqué

**Affiliations:** 1 CHU Orléans, Orléans, France; 2 SuLiSoM, Université de Strasbourg, Strasbourg, Alsace, France; 3 PRISME, Institut Bergonié, Bordeaux, Aquitaine, France

**Keywords:** cancer, grief, partner, review, sedation, spouse

## Abstract

**Background:**

The spouse is the most at risk of developing psychological consequences following the loss of a partner (anxiety, depression, complicated grief) compared to other family caregivers. The principal aim of this study was to investigate the possible implications and bereavement process for those who have lost a spouse following a cancer diagnosis and the implementation of continuous deep sedation (CDS).

**Methods:**

A scoping review was conducted according to the PRISMA protocol using the following databases: PubMed, Cochrane Library, Google Scholar, PsycInfo, Overview, PsycArticles, and PubPsych. The publication period used as a selection criterion was 2010–2023.

**Results:**

A total of 317 articles emerged from the keywords. However, the research studies focused exclusively on the practice of CDS, and the consequences of bereaved partners did not produce any results.

**Conclusions:**

The absence of selected articles has revealed various reflections and questions. Is it possible that CDS and its effects on grief are not evaluated because the practice is infrequent? As perceived and symbolically associated with euthanasia, can this lead to moral conflicts where it is illegal, and therefore generate a taboo? Given the results of the following study and the role of the grieving partner, it is essential to conduct further research on this topic and to suggest it.

## Introduction

Approximately 40 million people each year require palliative care, an approach that improves the quality of life of patients and their families. Palliative care aims to prevent and alleviate the pain perceived by the person diagnosed with a profoundly severe and generally terminal disease. Palliative care specialists assess and treat pain and other physical, psychosocial, social, or spiritual problems (World Health Organization, 2020).

Palliative care teams use an interdisciplinary approach, with professionals playing distinct roles: physicians, nurses, support workers, psychologists, paramedics, pharmacists, physiotherapists, and volunteers (World Health Organization, 2020).

According to the World Health Organization (2020), worldwide, 34% of patients who activate and seek palliative care have cancer, the globally second leading cause of death after cardiovascular diseases.

Furthermore, within palliative care regimes, in severe cases, Continuous Deep Sedation (CDS) is used to reduce the suffering caused by symptoms with a sedative effect to induce a deep state of unconsciousness until the moment of death (Bruinsma et al., 2014).

The international literature does not employ a single, universally accepted term to describe sedation practices at the end of life. Various expressions—such as continuous sedation, palliative sedation (PS), terminal sedation, continuous PS therapy, continuous deep sedation, or even slow euthanasia—are used interchangeably, often referring to overlapping but not identical practices (Papavasiliou et al., 2014; Rys et al., 2012; Rietjens et al., 2006; ten Have and Welie, 2014). This terminological heterogeneity complicates the comparison of studies and the identification of relevant evidence within the literature. For this reason, and to ensure clarity and consistency throughout the present article, we adopt the term Continuous Deep Sedation (CDS) to refer to the practice under examination.

Patients are, therefore, suffering from a severe, incurable, life-threatening disease with a short-term prognosis. Patients are presenting with so-called refractory suffering. This refractory suffering must be objectified by a third-party, recognized professional (Pierron, 2020). The High Authority of Health (Haute Autorité de santé) and the French Society of Support and Palliative Care (Société Française d'Accompagnement et de soins Palliatifs) recommendations specify the indications for applying this law (Haute Aut. Santé, 2020; Guirimand et al., 2010). The medications used, the implementation modalities, the technical decision-making tools, and the scales to evaluate the patient's comfort and the depth of sedation are described (Sessler et al., 2002).

Several studies in the scientific literature have examined professionals' subjective experiences when making decisions and establishing a CDS (Vieille et al., 2021; Morita et al., 2019; Camartin and Björkhem-Bergman, 2022; Belar et al., 2022; Abarshi et al., 2014; Lux et al., 2017). Physicians and nurses formulate the need to exchange with the patient and their relatives, anticipate sedation, and discuss with the different health actors (Chazot and Henry, 2016; Salas et al., 2020; Mesnage et al., 2020; Chemrouk and Geniey, 2020; Peyrat-Apicella and Chemrouk, 2022). The National Centre for Palliative and End-of-Life Care's (Centre national des soins palliatifs et de la fin de vie) survey on CDS (Louarn, 2017) shows that sedation is often proposed by the medical team and less frequently requested by the patient. According to Serey et al. (2019), the patient's main motive is a refractory symptom that corresponds to psychological suffering in most cases (69%). Health professionals express the complexity of these moments when the CDS is evoked and implemented.

Regarding families, most report satisfaction and view the treatment positively, particularly regarding the possibility of providing the patient with complete relief from symptoms (Tomczyk, 2018). Others, however, view CDS as emotional stress and discomfort, determined, for example, by the inability to communicate with the patient. They argue that CDS could accelerate death (Shen et al., 2018). Maintaining the bond can also take the form of physical contact. Yet, the longer continuous deep sedation lasts, the harder it becomes for relatives to manage the growing sense of emptiness. Several studies suggest that prolonged sedation increases family distress, with the waiting itself becoming a source of stress (Bruinsma et al., 2014; Raus et al., 2014). This suffering may even lead some families to wish to hasten their loved one's death.

As recalled by Areia et al. (2020), cancer and its treatments affect not only the patient but also the family caregivers, who are, in 85% of cases, spouses or adult children (Clark Paul et al., 2011). Furthermore, the entire social network surrounding the sick person is affected practically and emotionally (Areia et al., 2020).

It is estimated that about 20% to 50% of family caregivers of end-stage cancer patients suffer from adverse psychological sequelae (Areia et al., 2020), including distress, depression (Dionne-Odom et al., 2016; Guldin et al., 2012; Bacqué and Hanus, 2020; Holtslander et al., 2017), insomnia (Holtslander et al., 2017), anxiety (Areia et al., 2020; Rumpold et al., 2016), intensified burden (Areia et al., 2020), high levels of somatization (Dionne-Odom et al., 2016), anticipatory (Rumpold et al., 2016), and complicated grief (Dionne-Odom et al., 2016; Guldin et al., 2012; Bacqué and Hanus, 2020).

According to the literature, the predictors that could negatively influence the psychological course of the end of life correspond to the female gender (Areia et al., 2020), the mental and physical health status of the caregiver (Brazil et al., 2005; Rossi Ferrario et al., 2004), the social support received (Brazil et al., 2010), uncontrolled symptoms and pain, attachment to the patient (Bacqué and Hanus, 2020), and to the circumstances surrounding the death (Shear, 2015). Other factors contributing to bereavement complications include intrinsic factors related to the bereaved person's life course, personality, contextual factors related to the death process, communication with the health care team, and support received by the bereaved (Fasse et al., 2013; Zech, 2019). Furthermore, the risk of co-morbidity increases with the bereaved person's age, especially the presence of depression (Robbins-Welty et al., 2018), the risk of cardiovascular disease, and increased cognitive decline (Atalay and Staneva, 2020). Difficulties in the grieving process increase in cases of assisted suicide or euthanasia (Pott et al., 2011; Diricq, 2017; Ganzini et al., 2009).

Finally, the degree of affection between the bereaved and the deceased is a determining factor in the risk of grief complications, which is more significant following the loss of a partner than, for example, that of a parent or grandparent (Fernández-Alcántara and Zech, 2017; Garrouste-Orgeas et al., 2019; Prigerson et al., 2009). However, despite this notion, there are not many studies on the bereaved spouse in cancer (Rumpold et al., 2016; Fasse et al., 2014; Götze et al., 2014; Egerod et al., 2019; Aoyama et al., 2021). Most research has focused on the practices and attitudes of physicians (Bruinsma et al., 2014) and the consequences on general informal caregivers (Morita et al., 2004) and other family members (Areia et al., 2020). Little is known about the patient's partner and the effects that specific palliative interventions may have on their grief.

For this reason, this study aims to conduct a systematic literature review to investigate the potential consequences and bereavement process for individuals who have lost a spouse to cancer and have undergone deep and continuous sedation. We also aim to assess the quality of research in this field.

The results of this study will provide more information and need for consideration for all those involved in or working with this practice, including researchers, doctors, family caregivers, and healthcare professionals.

## Methods

To respond to the research objectives, a literature review was conducted according to the recommendations provided by the PRISMA protocol for scoping a review (Morita et al., 2004). The PRISMA method enables an exhaustive review of all the literature published in the central databases on a theme that will be defined from intersecting keywords.

The databases used were PubMed, Cochrane Library, Google Scholar, PsychInfo, Overview, PsycArticles, and PubPsych.

The selection period was limited to 2010–2023 to minimize conclusion bias based on prior findings and those irrelevant to contemporary research. The decision to limit the literature review to studies published from 2010 onwards was made deliberately to ensure that the findings are relevant and applicable to current research. By excluding studies conducted before 2010, the authors aimed to focus on recent advancements (Sani and Bacqué, 2023). They recognized that the rapid evolution of technology and therapeutic approaches in the past decade could make some earlier findings less relevant to the present context.

The research was carried out in English using the following search string: (“deep sedation” OR “continuous deep sedation” OR “palliative sedation”) AND (“partner” OR “spouse” OR “wife” OR “husband”) AND (“grief” OR “bereavement” OR “mourning”) AND “cancer”.

Two researchers (YC, LS) independently reviewed titles and abstracts identified starting from the keywords. Then, in pairs, the researchers independently screened the titles and abstracts of all articles retrieved. In a consultative procedure, consensus on which articles to screen full text was reached by discussion. Next, they independently screened full-text articles for inclusion.

The first selection occurred through the titles and abstracts of the potentially pertinent articles.

Subsequently, duplicates were discarded.

In addition to the studies related to the research objectives and the language of publication, two other inclusion criteria were used, namely:

(1) The studies were to be scientific articles.
(2) The articles had to appear in peer-reviewed journals.

Finally, all articles concerning children, adolescents, and other literature reviews were excluded.

Therefore, the selection was made by evaluating the topics dealt with in the articles without specifically selecting further criteria such as sensitivity or sensibility.

Starting exclusively from clinical experiences and perceiving missing information at the literature level, the aim was to quantify and present scientific articles published on the psychological consequences of those who have lost a partner following a cancer diagnosis and the activation of sedative practices in the field of palliative care. This analysis, therefore, concerns the presentation of the articles through the name of the authors, the objectives, the methodology, and the results obtained.

## Results

**Figure 1:**
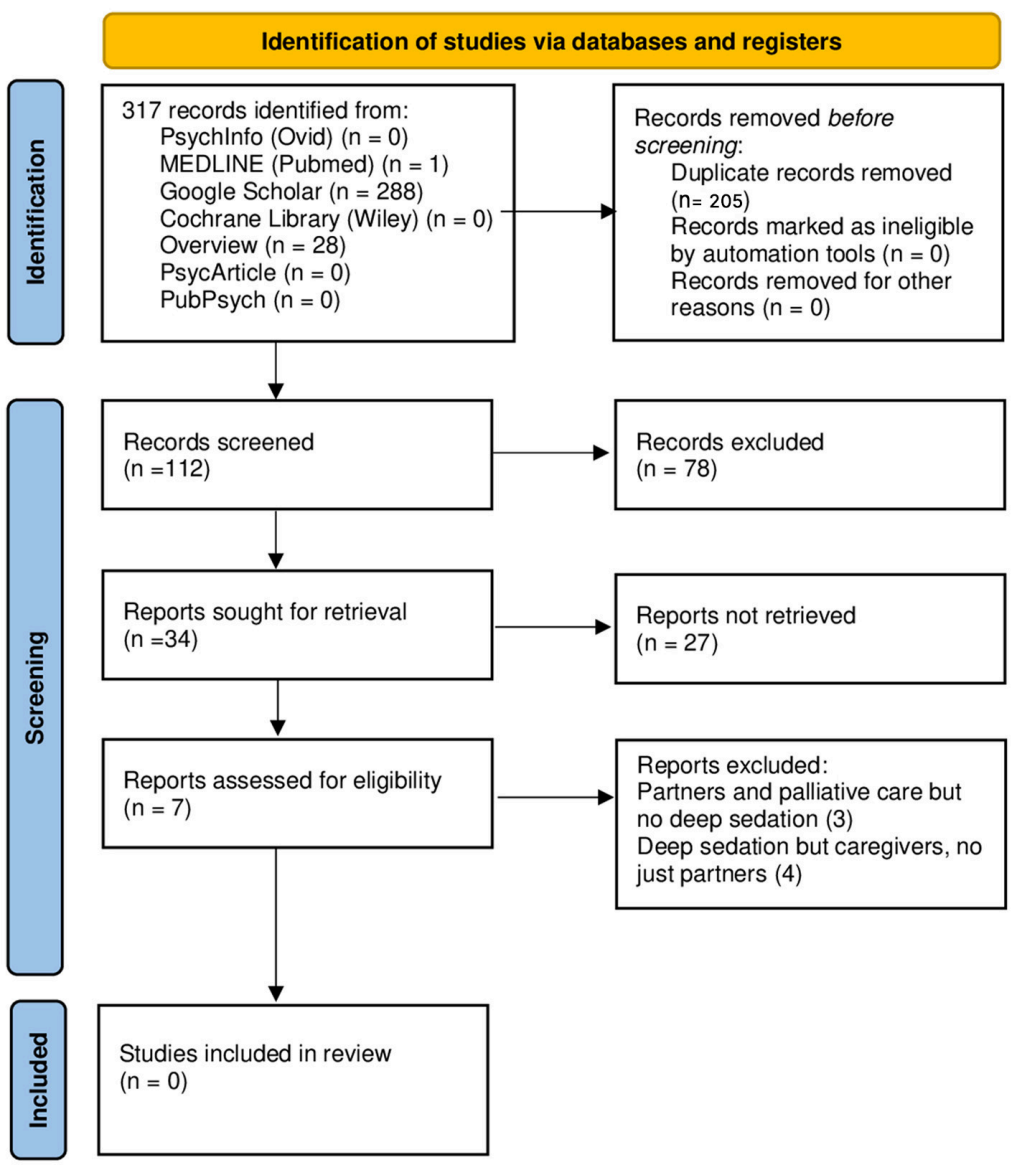
PRISMA flow diagram.

The literature review process is summarized in Figure 1.

Through the keywords listed above, the two authors analyzed 317 articles.

Starting from the comparison between the 288 articles found on Google Scholar, the 28 on Overview, and the article on PubMed, 205 were duplicates.

Table 1 shows the 34 articles selected based on their title and abstracts.

**Table 1:** The 34 articles selected by title and abstracts categorized according to their topic.

**First author (year)**	**Palliative care**	**Continuous deep sedation until death (CDSUD)**	**Spouse**	**General informal caregiver**
Aoyama et al. (2020)	Yes	No	No	Yes
Aoyama et al. (2021)	Yes	No	No	Yes
Aoyama et al. (2018)	Yes	No	No	Yes
Areia et al. (2019)	Yes	No	No	Yes
Bajwah et al. (2020)	Yes	No	No	Yes
Breen et al. (2020)	Yes	No	No	Yes
Bruinsma et al. (2014)	Yes	No	No	Yes
Bruinsma et al. (2016)	Yes	No	No	Yes
Chan et al. (2013)	Yes	No	No	Yes
Coelho et al. (2020)	Yes	No	No	Yes
Coelho et al. (2022)	Yes	No	No	Yes
Dionne-Odom et al. (2016)	Yes	No	No	Yes
Egerod et al. (2019)	Yes	No	Yes	No
Fasse et al. (2014)	Yes	No	Yes	No
Fringer et al. (2018)	Yes	No	No	Yes
Guldin et al. (2012)	Yes	No	No	Yes
Hamano et al. (2021)	Yes	No	No	Yes
Hayashi et al. (2021)	Yes	No	No	Yes
Hiratsuka et al. (2021)	Yes	No	No	Yes
Hirooka et al. (2018)	Yes	No	No	Yes
Holtslander et al. (2017)	Yes	No	No	Yes
Imai et al. (2022)	Yes	Yes	No	Yes
Jones et al. (2022)	Yes	No	Yes	No
Kagawa-Singer (2011)	Yes	No	No	Yes
Kissane et al. (2006)	Yes	No	No	Yes
Kissane et al. (2003)	Yes	No	No	Yes
Milberg et al. (2019)	Yes	No	No	Yes
Miyashita et al. (2017)	Yes	No	No	Yes
Shen et al. (2018)	Yes	Yes	No	Yes
Thomas et al. (2014)	Yes	No	No	Yes
Ullrich et al. (2020)	Yes	No	No	Yes
Ventura de (2016)	Yes	No	No	Yes
Wiese et al. (2010)	Yes	No	No	Yes
Zordan et al. (2019)	Yes	No	No	Yes

They are related to palliative care provided following a cancer diagnosis. The table indicates the authors and whether each article considers deep sedation, bereaved partners, or family members/informal caregivers.

Table 2 shows the percentages of 34 articles. Seven articles were chosen for eligibility. Three articles (8.8%) were found not dealing with deep sedation but with partners and palliative care (Fasse et al., 2014; Egerod et al., 2019; Jones et al., 2022), of which two were literature reviews (Fasse et al., 2014; Jones et al., 2022).

**Table 2:** Percentages of the topics of the 34 articles selected.

	***N***	**%**
Palliative care	34	100
Continuous deep sedation until death (CDSUD)	4	11.8
Partner	3	8.8
General informal caregiver	31	91.2
**Combination**		
Palliative care—continuous deep sedation until death (CDSUD)	4	11.8
Palliative care—partner	3	8.8
Palliative care—general informal caregiver	31	91.2
Continuous deep sedation until death (CDSUD)—partner	0	0
Continuous deep sedation until death (CDSUD)—general informal caregiver	4	11.8

Four other articles (14.3%) dealt with deep sedation for informal caregivers (i.e., spouses and children) (Bruinsma et al., 2014, 2016; Shen et al., 2018; Imai et al., 2022).

These articles demonstrate several interesting aspects concerning bereaved loved ones:

While most loved ones thought sedation had contributed to a “good death” for the patient, many expressed concerns. These included concerns about the patient's well-being, their well-being, and questions about whether continuous sedation had shortened the patient's life or whether another approach would have been better (Bruinsma et al., 2014).

This article compares practices in the Netherlands, Belgium, and the UK (Bruinsma et al., 2014).

In the Netherlands and Belgium, several relatives reported that the start of sedation had enabled them to plan a moment of “goodbye.” In contrast, British relatives did not discern an explicit start of sedation or a specific moment of farewell.

Most relatives reported being generally comfortable with the use of palliative sedation; nevertheless, a proportion of them experienced notable distress during the sedation period, highlighting the coexistence of acceptance and emotional burden (Bruinsma et al., 2016).

In a comparative study assessing relatives' experiences with continuous deep sedation until death vs. transient sedation (Bruinsma et al., 2016), no statistically significant differences emerged in the overall evaluation of the dying phase. Levels of concern, satisfaction, and perceived balance between symptom relief and preserved communication were comparable across groups. Conversely, several indicators of care quality—such as interactions with medical staff, nursing care, coordination of care, and procedural consistency—were rated significantly higher in the continuous deep sedation group. Overall, relatives' experiences of continuous deep sedation were not inferior to those associated with transient sedation and were, in some domains, evaluated more positively.

## Discussion

Using the six search engines previously described, the keywords generated an initial set of 317 articles. Considering the breadth of databases consulted and the 12-year time frame (2010–2023), this number appears relatively limited. As already mentioned, the selection process did not yield any studies specifically examining the psychological consequences experienced by bereaved partners after the use of continuous deep sedation (CDS). Although several studies have explored sedation practices or general family experiences, none addressed the particular situation of spouses—despite evidence identifying them as the relatives at highest risk for grief-related complications (Fernández-Alcántara and Zech, 2017; Garrouste-Orgeas et al., 2019; Prigerson et al., 2009).

Of the 34 articles retained after preliminary screening, only 4 (11.8%) addressed CDS (see Table 2). The scarcity of research on this topic is striking, especially given that partners are consistently reported to be the most vulnerable group in bereavement, notably in the context of cancer (Rumpold et al., 2016; Fasse et al., 2014; Götze et al., 2014; Egerod et al., 2019; Aoyama et al., 2021; Jadhav and Weir, 2018; Fagundes et al., 2019; Siflinger, 2017). This discrepancy suggests that, while bereavement in spouses has been investigated, the specific intersection between CDS and partner grief remains largely unexplored.

In reviewing the broader literature, several possible explanations emerge. First, research in palliative care rarely distinguishes between different categories of relatives: partners, adult children, and other family members are often treated as a single group, which may obscure the specific experiences of spouses (Bruinsma et al., 2014, 2016; Shen et al., 2018). Second, the variability in terminology and clinical practices surrounding sedation—ranging from “palliative sedation” to “terminal sedation,” “continuous deep sedation,” or even “slow euthanasia” (Papavasiliou et al., 2014; Rys et al., 2012; Rietjens et al., 2006; ten Have and Welie, 2014)—complicates literature searches and may contribute to inconsistent indexing of studies. This conceptual heterogeneity could partially explain why the search did not identify more targeted research.

Third, CDS may be used relatively infrequently in some healthcare contexts, making it challenging to collect sufficiently large samples to study bereavement outcomes in partners. In addition, the symbolic and ethical implications of CDS may discourage empirical investigation. As highlighted by Claessens et al. (2008), CDS may be perceived as closely related to euthanasia, particularly in countries where assisted dying is illegal. Such associations may create moral discomfort for clinicians and researchers, resulting in fewer studies addressing the psychosocial implications of sedation. Moreover, previous research indicates that while relatives often view CDS as contributing to a “good death,” they may also experience distress linked to communication loss, uncertainty, or unanticipated events during the dying process (Bruinsma et al., 2014; Peyrat-Apicella and Chemrouk, 2022; Raus et al., 2014). These emotionally complex situations may make it more challenging to recruit bereaved spouses for research participation.

Taken together, these findings suggest that the lack of studies on partners' bereavement after CDS is not merely an absence of data but a reflection of broader structural, methodological, and ethical challenges. This gap is clinically significant: given the heightened vulnerability of spouses, targeted research is essential to understand their experiences better, anticipate potential risks for complicated grief, and guide tailored support interventions in palliative care.

## Limitations

Finally, this article also presents some limitations. The methodology itself entails certain constraints. The scoping review approach was deliberately chosen to explore an under-researched topic and to map all potentially relevant evidence; however, it does not allow for a systematic assessment of study quality, a formal risk-of-bias evaluation, or a quantitative synthesis of results. Moreover, the absence of a universally recognized terminology for sedation practices, combined with heterogeneous clinical applications of CDS across countries, further limits the comparability of the retrieved studies.

At the same time, these limitations highlight an important strength of our work. The scarcity of studies identified through this comprehensive search points to a substantial and previously overlooked gap in the international literature. This gap underscores the need for further empirical research specifically addressing the experiences and psychological outcomes of bereaved partners of patients who underwent continuous deep sedation until death. The novelty of this topic underscores the relevance and timeliness of exploring it in greater depth.

## Conclusion

In conclusion, no articles emerged from the literature search concerning deep and continuous sedation until death and bereavement for spouses of cancer patients. It is essential to broaden research interests to the role and experience of the partner to better understand what is at stake for the relatives of a patient who has died under sedation. It is also advisable to disseminate this research on sedation, including other caregivers (formal and informal) who accompany the patient. Sedation and its symbolic and psychic effects have been evaluated in relatives, demonstrating a scientific and clinical interest in this hypothesis. Nevertheless, the effects of deep and continuous sedation until death on mourning, i.e., on the process considered a temporality, has not been studied.

## Data Availability

The original contributions presented in the study are included in the article/supplementary material, further inquiries can be directed to the corresponding author.
